# The many faces of precision (Replies to commentaries on “Whatever next? Neural prediction, situated agents, and the future of cognitive science”)

**DOI:** 10.3389/fpsyg.2013.00270

**Published:** 2013-05-21

**Authors:** Andy Clark

**Affiliations:** Department of Philosophy, University of EdinburghEdinburgh, UK

**Keywords:** prediction, predictive coding, hierarchy, embodiment, precision

## Abstract

An appreciation of the many roles of “precision-weighting” (upping the gain on select populations of prediction error units) opens the door to better accounts of planning and “offline simulation,” makes suggestive contact with large bodies of work on embodied and situated cognition, and offers new perspectives on the “active brain”. Combined with the complex affordances of language and culture, and operating against the essential backdrop of a variety of more biologically basic ploys and stratagems, the result is a maximally context-sensitive, restless, constantly self-reconfiguring architecture.

## Introduction

I am grateful to all the commentators for their friendly, challenging, and illuminating reactions to my foray into the world of probabilistic prediction machines. When I first drafted the target paper, I had no idea it would grow into a huge 2-year project, spawning more than 50 responses spread across two major journals. Nor could I predict how much I would need to learn (and sometimes unlearn) to even begin to approach these issues.

Reading and re-reading the many responses, I was struck by how many times my best response consisted—to a first approximation—in exploring the possible impact of a key mechanism that (see especially the comments by *Kiverstein and Reitveld*) was insufficiently stressed in my original treatment. That mechanism was the “precision-weighting” of prediction error. This provides a powerful means of inducing all manner of context effects, and may even hold the key to the orchestration of multiple different regions (and strategies) within the brain. Such precision-weighting amounts to altering the gain on select populations of prediction error units. This enables the flexible balancing of top-down and bottom-up influence, as described in the original paper. But it also provides a means of altering the flow of influence between different neural areas, hence flexibly reconfiguring patterns of effective connectivity. This, I shall argue, delivers a multi-purpose architecture in which response becomes just about maximally context-sensitive.

Another major shortfall of the original treatment was my failure to address the relations between rich, model-based prediction, and the superficially messy multitude of other elements underpinning cognitive and adaptive success. Here too, precision-based modulations of effective connectivity may prove important.

In what follows, I try to remedy both these shortcomings while responding to many (though by no means all) of the issues raised by the commentaries^1^. Biological agents emerge as *pro-active survival-enabled prediction machines*. These are prediction machines equipped with multiple ecologically sound routes to effective adaptive response, forever in the business of predicting their own unfolding sensory arrays. Within these machines patterns of effective neural (and extra-neural) connectivity are constantly in flux, in ways that both determine and are determined by their own actions, their affective and interoceptive states, and long-term goals.

## Precision, planning, and agency

Several commentators (*Boccignone and Cordeschi*; *Schilling and Rohlfing; Pezzulo; Conci Zellin, and Müller; Brown and Brüne; Brigard: Basso)* noted the need to accommodate forms of planning and offline reasoning that go far beyond the simple programming of here-and-now motor action^2^, and that sometimes seem to require something like the offline exploration of multiple possibilities. Here, the canny manipulation of precision-weighted prediction error may play a large role. A good trick (beautifully displayed in the comments from *Pezzulo*) is to re-use elements of the generative model that is used to produce our own actions to run simulations that allow us to explore possible courses of future action.

Thus, take some animal that commands a rich and powerful generative model enabling it to predict the sensory signal across many temporal and spatial scales. Such an animal seems well-placed to use that model “off-line” (see e.g., Grush, 1995, 2004; Clark and Grush, 1999) so as to imagine possible future unfoldings and select an action accordingly. But within the Predictive Processing (PP) framework, the deep intimacy of perception and action (see the original text) breeds a striking problem. For according to PP, thinking of a certain trajectory of arm motion (to take a very simple example) is the way to bring that trajectory about!

The solution (Friston et al., 2011) may lie in the canny deployment of precision-weighting. The proposal is that the brain, in order to simulate future unfoldings, must mute the weighting on select aspects of the proprioceptive prediction error signal. Suppose this is done while simultaneously entering a high-level neural state whose rough-and-ready folk-psychological gloss might be something like “I reach for the cup.” Motor action, on the PP account, is entrained by proprioceptive expectations and cannot here ensue. But all the other intertwined elements in the generative model remain poised to act in the usual way. The result should be a “mental simulation” of the reach and hence an appreciation of its most likely consequences. Such mental simulations provide an appealing way of smoothing the path from basic forms of embodied response to abilities of planning, deliberation, and “off-line reflection”—see Barsalou (1999, 2009); Grush (2004); Pezzulo (2008); Pezzulo et al. (2013)—see also Hesslow (2002); Colder (2011). They may also shed light (see the remarks by *de Ridder, Verplaetse, and Vanneste*) on the complex issues concerning the experience of free will. Agents that can, for example, simulate multiple motoric unfoldings are well-placed to feel that any one of those could become actual “at will.”

In simulation, as *Brown and Brüne*, and *Schilling and Rohlfing*, nicely suggest, we may also re-use the knowledge that drives our own motor responses as a means of *simulating and understanding the actions of other agents*. Within the PP framework, this could come about in much the same way as any other form of “offline simulation.” Thus, given some cues that inform me that I am watching another agent, the precision-weighting (the gain) on proprioceptive prediction error relative to those aspects of the observed scene should again be muted^3^. With the gain on proprioceptive prediction error turned down, we are free to deploy the generative model geared to the production of our own actions as a means of predicting and understanding the actions of others. Under such conditions, the complex interdependencies between other aspects of the generative model (those relating high-level aims and intentions to proximal goals and to the shape of the unfolding movements) remain active, allowing prediction error minimization across the cortical hierarchy to settle on a best overall guess concerning the intentions “behind”^4^ the observed behavior.

The upshot is that: “We can use the same generative model, under action or observation, by selectively attending to visual or proprioceptive information (depending upon whether visual movement is caused by ourselves or others)” Friston et al. (2011) (p. 156).

By contrast, when engaged in self-generated action, the precision-weighting on the relevant proprioceptive error must be set high. When proprioceptive prediction error is highly weighted yet suitably resolved by a stack of top-down predictions (some of which reflect our goals and intentions), we feel that we are the agents of our own actions. Core aspects of the much-discussed “sense of agency”^5^ (see the comments by *White and Shergill* and by *Kumar and Srinivasan*) seem to depend upon this, and mistakes in both the generation of prediction errors and the assignment of precision-weighting to such errors are increasingly though to underlie many illusions of action and control [as nicely displayed in the comments from *White and Shergill*, and see, for example, Fletcher and Frith (2009); Friston (2012); Ford and Mathalon (2012)].

*Kumar and Srinivasan* suggest a “hierarchical event control” framework (see Jordan, 2003) as a possible means of implementing related ideas, but one that supports a more dynamic and fluid account of the experience of agentive control. The key idea, if I understand them correctly, is to allow the experience of agency to “attach” to different control loops according to an agent's expertise, local goals, and the changing effects of context. This would determine whether we feel in control of the car, or the gearbox, or our legs and hands, and so on. This image of a “shifting sense of agentive control” seems right, and it raises the question of exactly how salient control loops are selected and highlighted in ongoing experience. From within PP the answer, I suspect, must again involve to the weightings given to prediction error responses occurring in various places within the processing hierarchy: weightings that must shift and alter in ways that reflect our goals, expertise, and the context of action. I return to this issue in the next section.

To sum up, the neural representations that underlie our own intentional motor actions, those that underlie our simulations of possible future actions, and those that are active when we model the motor behavior of other agents may be substantially overlapping. Clear differences in functionality are here traced not to the core representations but to the estimations of precision that nuance their effects, reflecting the different contexts in play. If this is true, then:

“the brain does not represent intended motor acts or the perceptual consequences of those acts separately; the constructs represented in the brain are both intentional and perceptual [having] both sensory and motor correlates” Friston et al. (2011) (p. 156).

Such representations are essentially amodal, high-level associative complexes linking goals and intentions to sensory consequences. Those states have differing constellations of modality-specific implications (some proprioceptive, some visual, etc.) according to the context in which they occur: implications that are implemented by varying the precision-weighting of different aspects of the prediction error signal.

Systems like these combine a real sensorimotor grip on dealing with their worlds with the emergence of higher-level abstractions that (crucially) develop in tandem with that grip. This is because learning here yields representational forms, at higher processing levels, that allow the system to predict the regularities that are governing the neural patterns (themselves responding to energetic stimulations at the sensory peripheries) present at the lower levels. What emerges are “grounded abstractions” (for this general notion, see Barsalou, 2003; Pezzulo et al., 2013) that may open the door to more compositional and strategic operations, such as solving novel motor problems, mimicking the observed behavior of other agents, engaging in goal-directed planning, and pre-testing behaviors in offline imagery (For persuasive suggestions concerning the more complex forms of “cognitive control” that some of these require, see the comments from *Pezzulo*).

This whole emerging complex of ideas concerning action production, action understanding, and the capacity to simulate future events has been studied, in microcosm, using a variety of simulations. Weber et al. (2006) describe a hybrid generative/predictive model of motor cortex that provides just this kind of duplex functionality. In this work, a generative model that enables a robot to perform actions doubles as a simulator enabling it to predict possible chains of perception and action. This simulation capacity is then used to enable a simple but challenging behavior in which the robot must dock at a table in a way that enables it to grasp a visually detected object.

Related ideas are pursued by Tani et al. (2004), and by Tani (2007). Tani and colleagues describe a set of robotic experiments using “recurrent neural networks with parametric biases” (RNNPBs): a class of networks that implement prediction-based hierarchical learning. The guiding idea, again shared with PP, is that prediction-based hierarchical learning here solves a crucial problem. It allows a system to combine a real sensorimotor grip on dealing with its world with the emergence of higher-level abstractions that (crucially) develop in tandem with that grip. This is because learning yields grounded abstractions: representational forms, at higher processing levels, that allow the system to predict the regularities that are governing the neural patterns (themselves responding to energetic stimulations at the sensory peripheries) present at the lower levels.

Such grounded abstractions do not float free of their roots in embodied action. Instead, they constitute what might be thought of as a kind of “dynamical programming language” for those interactions: a language in which, for example, “continuous sensory-motor sequences are automatically segmented into a set of reusable behavior primitives” (Tani, 2007, p. 2). Tani et al. (2004) show that robots equipped (as a result of learning-driven self-organization) with such primitives are able to deploy them so as to imitate the observed behavior of another. This set of studies is further extended in Ogata et al. (2009), who tackle the important problem of viewpoint-translation using an RNNPB simulation in which one robot views and then imitates the object-manipulation behavior of another agent, applying a set of learnt transformations to its own self-model. In this experiment in “cognitive developmental robotics”:

“The other individual is regarded as a dynamic object that can be predicted by projecting/translating a self-model” Ogata et al. (2009) p. 4148.

Such demonstrations, though restricted in scope, are revealing. The emergence of “reusable behavior primitives” shows that features such as compositionality, re-usability, and re-combinability (features long associated with the more brittle symbol-structures of classical Artificial Intelligence) can arise quite naturally as a result of prediction-driven hierarchical learning. Combined with the development of viewpoint conversion capacities, this illustrates the way multi-level self-based predictions may come to be exploited in a much wider range of cases. Perhaps most importantly of all, however, the experiments show that multi-level prediction-based learning results in re-combinable, re-useable “abstractions” of a rather special kind: abstractions that remain richly grounded in the sensorimotor dynamics (and more broadly, in the past experience) of the agent, sharing in “the same metric space of physical dynamical systems” (Tani, 2007, p. 2).

## Sculpting effective connectivity

The shifting sense of agentive control described by *Kumar and Srinivasan* is important and phenomenally real. It may be related, I suspect, to another important precision-dependent effect. This is the effect of *sculpting patterns of effective connectivity* within the brain.

“Effective connectivity” (Aertsen et al., 1987; Friston, 1995—see also Horwitz, 2003; Sporns, 2010) names “the influence one neural system exerts over another” (Friston, 1995, p. 57). It is to be distinguished from both structural and functional connectivity. “Structural connectivity” names the gross pattern of *physical linkages* (the web of fibers and synapses) that—perhaps working in concert with more diffuse “volume signaling” mechanisms (see e.g., Philippides et al., 2000)—allow neurons to interact across space and time. “Functional connectivity” describes observed patterns of *temporal correlation* between neural events. The closely related notion of “effective connectivity” then aims to reflect short-term patterns of *causal influence* between neural events, thus taking us beyond simple observations of undirected—and sometimes uninformative—correlation. One quite useful way to think about the relation between functional and effective connectivity is to conceive of:

“the [electrophysiological] notion of effective connectivity … as the experiment and time-dependent, simplest possible circuit diagram that would replicate the observed timing relationships between the recorded neurons” (Aertsen and Preißl, 1991, quoted in Friston, 1995), p. 58.

Functional and effective connectivity patterns alter rapidly as we perform our cognitive tasks, with changes occurring within hundreds of milliseconds. Structural change, by contrast, is a slower process since it is, in effect, reconfiguring the reconfigurable network itself (by altering the underlying communicative skeleton that supports other, more rapid, forms of momentary reconfiguration).

Within the PP framework, the control of effective connectivity is achieved by the manipulation of the precision-weighting assigned to specific prediction errors. The primary effect of this is to systematically vary the relative influence of different neural populations by increasing the gain (“volume”) on selected error units. This offers a promising means of implementing fluid and flexible forms of large-scale gating^6^ among cortical populations. To see this, we need only note (once again) that very low-precision prediction errors will have little or no influence upon ongoing processing, and will fail to recruit or nuance higher-level representations. Altering the distribution of precision-weightings thus amounts, in effect, to altering the “simplest circuit diagram” (Aertsen and Preißl, 1991) for current processing. Our shifting sense of agentive control, it might be speculated, tracks (when all is working correctly) changes in these simplest circuit diagrams—see also the comments from *Kumar and Srinivasan*. This suggests a crucial departure (see the comments from *Boccignone and Cordeschi*) from the image of a simple, fixed, hierarchical organization in the brain. Insofar as patterns of effective connectivity can be rebuilt “on the fly,” we do not confront a simple fixed hierarchy so much as a flexible, reconfigurable flow. Such flows are defined over an underlying bi-directional hierarchical organization. But we are here exploring territory hugely distant from the classical vision of a static, serial, feed-forward hierarchy.

The neural mechanisms of attention, PP suggests, are thus identical with the neural mechanisms that alter patterns of effective connectivity. This is an intuitive result (see also Van Essen et al., 1994), especially if we consider that the specific means by which such alterations may be effected are many, and that their detailed functional implications may vary in different parts of the brain. Possible implementing mechanisms for the precision-weighting of prediction error (which, in PP, amounts to the control of post-synaptic gain) include the action of various “modulatory neurotransmitters” such as dopamine, serotonin, acetylcholine, and noradrenalin (Friston, 2009). Frequencies of oscillation (for an excellent window onto the complexities hereabouts, see the comments by *Yordanova, Kolev, and Kirov*) must also play a major role—see Engel et al. (2001); Hipp et al. (2011). These mechanisms also interact, since [to take just one example, from Feldman and Friston (2010)] gamma oscillations respond to acetylcholine. Thus, while the notion of sculpting patterns of effective connectivity by means of “precision-weighted prediction error” is simple enough, the mechanisms that implement such effects may be multiple and complex, and they may interact in important but as yet under-appreciated ways.

Support for this general idea (the idea of prediction-error-based reconfiguring of large-scale patterns of effective connectivity) was recently provided by an fMRI study analysed using non-linear Dynamical Causal Modeling (Friston et al., 2003). In this study (den Ouden et al., 2010) specific prediction error signals in one (striatal) neural area modified the coupling between other (visual and motor) areas. Failures of prediction (caused by changing contingencies within the experimental setting) systematically altered the strength of the visuomotor coupling in a way that was “gated by the degree of prediction error encoded by the putamen” (den Ouden et al., 2010, p. 3217). This is an important result, demonstrating that:

“trial-by-trial prediction error responses in a specific region modulate the coupling among other regions” den Ouden et al. (2010), p. 3217

Here, the amount of striatal prediction error delicately controls the strength (efficacy) of the visuomotor connection. The context-varying interplay between visual and motor regions is thus orchestrated by striatally computed prediction error (this is in line, I think, with the comments on the importance of the striatum by *Bernacer and Murillo* and may offer another angle upon some of *Spurrett*'*s* helpful suggestions concerning the roles for prediction error computations elsewhere in the brain).

All this yields a picture of neural dynamics that is (as rightly demanded in the probing commentary from *Kiverstein and Rietveld*) just about maximally sensitive, at multiple time-scales, both to varying task-demands and to the estimated reliability (or otherwise) of specific bodies of top-down expectation and bottom-up sensory input. Information about tasks, about our own interoceptive and emotional states (see the comments by *Moore*, by *Bernacer and Murillo*, and by *Roesch, Nasuto, and Bishop*) and about local context thus recruit not just a set of predictions but (crucially) a *set of predictions that include precision-expectations*. Such expectations enable an inner organization that is dynamically self-reconfiguring in ways that respond to tasks, background state, background knowledge, and current environmental affordances.

Downwards-flowing influence here has a major modulatory impact on the selectivity of lower-level response, so that activity that (in one context) correlates with one external state of affairs may (in another context) become tuned so as to respond to something different (for some examples, see Friston and Price, 2001). The brain thus construed is “labile” and comprises “an ensemble of functionally specialized areas that are coupled in a nonlinear fashion by effective connections” (Friston and Price, 2001, p. 277). Neural representations here become “a function of, and dependent upon, input from distal cortical areas” (Friston and Price, 2001, p. 280). This is a potent source of flexibility since the flow of input from such areas is *itself* subject to rapid restructuring by prediction error signals elsewhere in the brain.

When all these features combine, the result is an architecture in which there are distinct components but whose constantly shifting dynamics are [to borrow a phrase from Spivey (2007)—and see also Anderson et al. (2012)] “interaction dominated.” The highly negotiable flows of influence thus constructed are themselves action-responsive, leading to various forms of “circular causation” linking perception and action (see below) so the space of dynamical possibilities is further enriched by all manner of bodily and worldly tricks for structuring our own inputs and restructuring problem spaces.

## Multiple neural strategies

The tools are now on the table to begin to address a major issue that arose in different ways in many of the commentaries (see especially the comments by *Huebner*; *McBride*; *Calvo, Symons, and Martin*; *Moore*; *Nanay*; and *Sheredos*). This is the issue of multiple neural (and perhaps extra-neural—see below) mechanisms and strategies contributing to adaptive and cognitive success. Here too, the PP story benefits from the delicate use of precision-weighting as a means of altering patterns of effective connectivity.

It is common, for example, to distinguish between what are sometimes called (see e.g., Doya et al., 2002; Dayan and Daw, 2008; Dayan, 2012) “model-based” and “model-free” approaches to choice and decision-making. Model-based strategies rely, as the name suggests, on a model of the domain that includes information about how various states (worldly situations) are connected, thus allowing a kind of principled estimation (given some cost function) of the value of a putative action. Such approaches involve the acquisition and the (computationally challenging) deployment of fairly rich bodies of information concerning the structure of the task-domain. Model-free strategies, by contrast, “learn action values directly, by trial and error, without building an explicit model of the environment, and thus retain no explicit estimate of the probabilities that govern state transitions” (Gläscher et al., 2010, p. 585). Such approaches implement “policies” that associate actions directly with rewards, and that typically exploit simple cues and regularities while nonetheless delivering fluent, often rapid, response.

Model-free learning has been associated with a “habitual” system for the automatic control of choice and action, whose neural underpinnings include the midbrain dopamine system and its projections to the striatum, while model-based learning has been more closely associated with the action of cortical (parietal and frontal) regions (see Gläscher et al., 2010). Learning in these systems has been thought to be driven by different forms of prediction error signal—affectively-salient “reward prediction error” (see e.g., Montague et al., 1996; Schultz et al., 1997; Hollerman and Schultz, 1998) for the model-free case, and more affectively neutral “state prediction error” for the model-based case. These relatively crude distinctions are, however, now giving way to a much more integrated story (see e.g., Daw et al., 2011; Gershman and Daw, 2012) as we shall later see.

PP itself is, as several commentators noted, a claim concerning a fundamental model-based strategy^7^ that may be at work throughout motor and sensory cortex. That strategy involves the use of ongoing attempts at self-prediction to drive the development (on-the-hoof) of a rich, multi-layer generative model capturing interacting hidden causes spanning multiple spatial and temporal scales. That same generative model, we have seen, may then be used in perception, action-production, and for the simulation of action (our own, or that of other agents). How, then, should we conceive the relations between this story and the large literature on model-free learning and reward prediction error?

Given that the brain is always active, and that we encounter new scenes with rich contextualizing expectations already in play, the use of a rich, cortically-represented generative model is perfectly consistent with the production of rapid, “cheap” (i.e., involving relatively few processing steps) responses. That means there can be no direct inference from speedy, fluent, response to the claim that some model-free strategy is in play. Despite this, we need not (and should not) suppose that *all* our responses and cognitive operations depend on inferences grounded in a rich, cortically-represented (probabilistic) internal world model. Instead, we may often rely on quick-and-dirty strategies such as the deployment of “cached” (in effect pre-computed—see Daw et al., 2005) solutions. Such solutions need not be seen as sidestepping the cortical generative model so much as productively altering the conditions for its deployment. Action itself may often play such a role, as when we shift our gaze so as to enable a simpler cue to control a complex action. Action-recruiting loops like these are thus mandated by the generative model itself. But their effect is to alter the problem space so as to allow simple, rapidly processed cues to deliver apt world-engaging actions [such, in microcosm, seems to me to be the nature of much human (and non-human) expertise].

This opens up an interesting possibility. For an under-appreciated role for precision-expectations (one directly consequent upon the role of precision-weighting in sculpting effective connectivity) may be to arbitrate between the use of different neural strategies. Precision assignments reflect uncertainty, and provide (as we saw) a general tool for the context-sensitive manipulation of patterns of effective connectivity. If we suppose (over-simplistically—see below) that there exist multiple, competing neural resources capable of addressing some current problem (say, the need to decide on a course of action), there needs to be some mechanism that arbitrates between them. With this in mind, Daw et al. (2005) describe a broadly Bayesian “principle of arbitration” whereby estimations of the relative uncertainty associated with distinct “neural controllers” (e.g., “model-based” vs. “model-free” controllers) allows the most accurate controller, in the current circumstances, to determine action and choice. Within the PP framework this would be implemented using the mechanisms of precision-estimation and precision-weighting described earlier. Each resource would compute a course of action, but only the most reliable resource (the one associated with the least uncertainty when deployed in the current context) would get to determine high-precision prediction errors of the kind needed to drive action and choice. In other words, a kind of meta-model (one rich in precision expectations) would be used to determine and deploy whatever kind of strategy is best in the current situation.

This broad notion has much to recommend it. It provides hints of one way to begin to erode the worry (as pressed, from various directions, in the comments by Blokpoel, *Kwisthout, and van Rooij: Huebner*; Spurrett; *Sheredos; Calvo, Symons, and Martin; McBride*; and *Nanay*) that PP over-emphasizes a computationally expensive, sometimes intractable, representation-heavy strategy over other (quicker, dirtier, perhaps striatally computed) ones that more directly associate actions and rewards. Perhaps we deploy multiple strategies in ways adjudicated by a kind of Bayesian meta-model. This also helps to address the worry that the PP account sees the main task of brains as *representing* the world, rather than as selecting actions. On the contrary, PP puts action in the driving seat since it is only by means of action that the prediction error associated with a current perceptual state can be reduced.

The “model-based/model-free” distinction is intuitive, and resonates (for better or worse) with old dichotomies between habit and reason, and between emotion and analytic evaluation. But (whatever we make of that) the image of underlying, computationally distinct, parallel, neural sub-systems may not stand the test of time. A recent fMRI study (Daw et al., 2011) suggests that rather than thinking in terms of distinct model-based and model-free learning systems, we may need to posit a “more integrated computational architecture” (Daw et al., 2011, p. 1204) in which the different brain areas most commonly associated with model-based and model-free learning (the pre-frontal cortex and the dorsolateral striatum, respectively) *each* trade in both model-free and model-based modes of evaluations and do so “in proportions matching those that determine choice behavior” (Daw et al., 2011, p. 1209)^8^. In particular, they found that:

“even the signal most associated with model-free RL [reinforcement learning], the striatal RPE [reward prediction error], reflects both types of valuation, combined in a way that matches their observed contributions to choice behavior” Daw et al. (2011), p. 1210.

Such results demand a thorough reworking of the standard decision-theoretic model that posits distinct representations of utility and probability. In its place we will find something more nuanced: an integrated architecture in which:

“Perception, action, and utility are ensnared in a tangled skein [involving] a richer ensemble of dynamical interactions between perceptual and motivational systems” Gershman and Daw (2012), p. 308.

Top-down information, Daw et al. (2011) suggest, might here control the way different strategies are combined in differing contexts for action and choice. Within such an architecture, precision estimations must play a major role in enabling the kinds of metacontrol needed to negotiate the tangled skein.

Reflection upon strategies of rational (Bayesian) metacontrol will be essential, I suspect, if we are to determine how best to combine the PP model with neuroeconomic work on reward, as stressed by *Spurrett* and by *Huebner.* At that point, the rather large question whether cost functions can indeed be fully absorbed into prior beliefs about our future exchanges with the world should also become easier to resolve^9^.

## Language and the social spiral

*Blokpoel, Kwisthout, and Van Rooij* dispute the claim that PP implements a tractable form of Bayesian inference. The most that might be claimed, they argue, is that the inference is tractable (and Bayesian) so long as the structure of the model is constrained, exhibiting only what they describe as an “intermediate” level of causal complexity. The basic point that Blokpoel et al. make must be conceded. There is no guarantee that the approximations required to make the PP strategy computationally tractable will always be available. For basic perception, apt (probably quite generic) constraints may be present courtesy of evolution, just as Blokpoel et al suggest. The puzzle really bites with higher—level forms of thought and reasoning^10^.

How do we humans manage to negotiate so many apparently hugely complex cognitive domains? I think the answer must lie in the canny re-use of the fruits of basic sensorimotor learning, and the iterated interactions of language and culture. We marinate human brains in a succession of artificially structured environments that constrain the causal models that the brain must learn, building gradually and incrementally upon our basic sensorimotor grip on reality.

We saw (section Sculpting Effective Connectivity) how we might re-purpose our own basic world models so as to understand others, and to simulate future unfoldings. This provides one way of scaffolding a representational trajectory through an otherwise dauntingly large space. Add to this the iterated interactions of language and culture. Such interactions allow us (both individually and as a species) to repeatedly recode complex problems so as to address them in new, lower dimensional forms (see Clark and Thornton, 1997; Clark, 1998, 2008). In this vein, *Lupyan* nicely depicts some of the benefits of “language-augmented prediction,” pointing to the role of language as a tool for cueing top-down predictions, and (especially) as a device for efficient category cueing. Language, Lupyan's experimental work shows, interacts deeply with perception, and does so in many ways that go far beyond the immediate effects of simple self-directed rehearsal (as when we rehearse a verbal formula for tying our shoelaces). Words in the environment act as powerful category cues, and as such help create the external scaffoldings necessary for many of our social, legal, and commercial institutions. Language (once mastered) also provides a cheap and flexible means for self-cueing, and this may be important for “programming” the kinds of simulations required to explore, and keep properly distinct, future options. Such language-based self-cueing would also recruit precision expectations, hence providing a cheap, easily agent-available, means of altering our own patterns of effective neural connectivity.

Culture (cultural practices, and the larger settings of schools, institutions, etc.) adds further layers of empowering complexity. Acting in concert with the multiple effects of linguistic encoding, such settings determine new regimes of statistical bombardment that install generative models whose reach and scope regularly exceed the reach and scope of those that came before. The upshot is that the goalposts for human prediction-based learning are constantly moving. Our cultural and linguaform resources thus complement (as *Newsome* convincingly argues) but need not replicate the benefits of neural prediction-based learning.

Language also provides, as Dennett (1991) famously notes, an expressive tool that enables us to depict our own mental states as more fully determinate than perhaps they actually are. I thus found myself I full agreement with Madary's interesting comments, and would only add that his idea of “indeterminate implicit anticipations” in perceptual experience might be augmented by the idea (Spivey, 2007) that our underlying neural states may not follow a sequence of attractors, settling first into one then another, so much as repeatedly approach them, spinning-off both linguistic and non-linguistic actions (each of which can make our mental states seem more determinate and unequivocal than they really are) along the way. All this fits nicely with the dynamical image of a meta-stable neural economy characterized by so-called “heteroclinic cycles”—see Friston et al. (2012a).

Our capacities to use language as a tool for self-manipulation may also increase the *appearance* (as noted by *Brigard*) of a deep disconnect between agent-level cognition and its probabilistic underpinnings. For language may act, within the larger probabilistic economy, as a kind of double-edged sword. On the one hand, language provides expressive resources enabling us to generate compact models of the world: models that include many layers of causal structure, capturing events, and processes at time-scales beyond the reach of non-linguistic creatures (and enabling, I suspect, many of the high-level planning capacities neatly described by *Basso*). On the other hand, language allows us to artificially prime our own responses in ways that often (see e.g., Kahneman, 2011) lead us astray, even in cases where the right answers are (if only the questions are posed in the right way) actually well within our reach. It may thus be this potent capacity for cheap, linguistically-mediated re-coding, priming, and self-priming that best explains both the power of and depth of human world model, and the many failures of conscious^11^ reasoning highlighted by *Perruchet and Poulin-Charronat.* The idiosyncrasies of linguistically-inflected cognition may also bear on Huebner's challenge concerning the origins of radical social change. The key issue there is not reward, it seems to me, but what might be described as “knowing engagement with own models of the world.” This notion [Dennett (2000) calls it “florid representing”] requires our own world-models to become objects of our thought, not just the means by which thought occurs. Language, I have argued (Clark, 1998), seems well-placed to aid such a process, since it makes inspectable public objects (written and spoken sentences) out of our own cognizings.

The effects of our massive exposure to the forms and structures of public language are likely to be extensive. In particular, I would speculate that language provides a powerful means of driving what Annette Karmiloff-Smith (1990—see also Clark and Karmiloff-Smith, 1993) calls “representational re-description”: the process whereby early, successful representational forms become repeatedly re-coded, in ways that support the integration of multiple bodies of knowledge and yield more powerful generalizations. Exactly how public linguaform encodings interact with the kinds of probabilistic knowledge representation posited by PP remains largely unknown, and constitutes an important target for future research.

## Bacteria, oil drops, and us

*Sheredos* notes that many key features of the PP account seem to apply to bacteria too. *Roesch et al.* note that even an oil drop can “solve” a maze puzzle courtesy of basic forms of structural coupling, and ask whether similar (i.e., “cheap” and “representation-lean”) strategies might not play a large role in linking embodied agents to their environments. *McBride* draws our attention to the role of fundamental, core processes of homeostatic regulation. *Moore* notes the importance of motivated, affectively-loaded routes to rapid situational response. *Calvo et al.* note the crucial role of morphology in enabling fluid world-engaging action. In different ways, each of these picks out elements and strategies that play crucial roles in securing adaptive success. It was never my intention to downplay these roles (and many of them are pursued in my own work: see Clark, 1997, 2008). Ignoring them threatens, as Nanay suggests, to over-intellectualize^12^ large swathes of adaptive response in both human and non-human animals. Adaptive success must indeed depend on complex admixtures of strategies including the canny use of bodily form and various “representation-lean” ploys.

Within that complex adaptive nexus, however, the kind of multi-level probabilistic prediction machinery described by PP plays (I claim) a doubly special role. First, it is plausibly only courtesy of that machinery that we are able to *experience a structured external world* (a world built of nested interacting distal causes) at all. The bacteria, if this is right, responds to its world but it does not truly know it. Second, that same machinery may be used to *arbitrate between the use of various available strategies*, including some that are “model-free.” Such strategies may not require segregated neural representations, but may be better treated as differing modes within a more integrated architecture. Either way, the use (when ecologically apt) of simple cues and quick-and-dirty heuristics is not just compatible with prediction-based probabilistic processing: it may also be *actively controlled* by it.

### Conflict of interest statement

The author declares that the research was conducted in the absence of any commercial or financial relationships that could be construed as a potential conflict of interest.

## Footnotes

^1^But see also the Author's Reply appearing after the target paper in *Behavioral and Brain Sciences*, where many additional issues are discussed.

^2^Both Conci et al. and Brigard are especially concerned with effects spanning multiple temporal scales. Brigard offers a compelling sketch of how to integrate memory into a probabilistic prediction-based framework and I fully agree that there is an illuminating potential “fit” hereabouts. Such a fit depends crucially upon context effects working at multiple temporal scales, as also highlighted in the useful suggestions by Conci et al. Brigard also raises the issue of agent-based vs. sub-personal frequency judgments. This bears on the more general question of the genesis and nature of the agentive perspective itself, and identifies an important area requiring both conceptual clarification and further empirical research.

^3^The suggestion here is not that our somatosensory areas themselves are rendered inactive during action observation. Indeed, there is considerable evidence [reviewed in Keysers et al. (2010)] that such areas are indeed active during passive viewing. Rather, it is that the forward flowing influence of (the relevant aspects of) proprioceptive prediction error now becomes muted, rendering such error unable to impact higher levels of somatosensory processing. Just such a pattern (of lower-level activity combined with higher level inactivity) was found—as Friston et al. (2011), (p. 156) point out—by Keysers et al. (2010). I use the word “muted” advisedly, having spoken (in an earlier version) of setting such weights at or close to 0. But full zero settings, as Shimon Edleman (pers. communication) usefully notes, can have dramatic consequences within finely balanced dynamical systems, giving rise to significant computational challenges.

^4^It may be worth noting that the intentions of other agents are no *more* hidden, on these accounts, than any other aspects of the scene before us. I infer your intentions in the same way, and using the same broad apparatus, as I infer that there is, in front of me, a yellow coffee cup on a wooden table.

^5^There is large and complex literature on this topic – see de Vignemont and Fourneret (2004) for a useful review, and some important fine-tuning of the basic notion. See also Hohwy (2007); Friston et al. (2012b).

^6^Neural gating hypotheses come in many forms, including the postulation of special populations of information-routing “control neurons” (Van Essen et al., 1994), the canny use of reentrant processing (Edelman and Mountcastle, 1978; Edelman, 1987) and the development of “convergence zones” (Damasio and Damasio, 1994). The latter are essentially hubs in which many feedback and feedforward loops converge, and which are thus able to “direct the simultaneous activation of anatomically separate regions” (Damasio and Damasio, 1994 p. 65). Within the PP framework, gating is achieved by the manipulation of the precision-weighting assigned to specific prediction errors.

^7^The “model-based/model-free” distinction, as Friston (pers. communication) notes, is not really relevant to his larger “free energy minimization” account, which fluidly covers both cases.

^8^This makes functional sense since model-free schemes may rely upon model-based schemes to teach them how to respond. This results in a hierarchical embedding of the (shallow) model-free responses in a (deeper) model-based hierarchy (a hierarchical generative model). Model-based schemes are also necessarily context-sensitive, unlike model-free or habitual schemes that—once in place—are context bound. In such cases inferential machinery identifies the appropriate contexts in which to deploy the model-free (“habitual”) schemes.

^9^For the full absorption model, see Friston et al. (2012c), and for some worries about that model, see Gershman and Daw (2012) pp. 304–306.

^10^The example they give is understanding others (i.e., Theory of Mind). Some of the resources available to address this case are discussed in section Precision, Planning, and Agency above.

^11^It strikes me as unlikely, despite the interesting comments by *Perruchet and Poulin-Charronat*, that the bulk of our internal representational activity is conscious. But an overall winning hypothesis (in perception, the multi-scale understanding that best predicts the sensory data) is thereby poised to exert a special grip on subsequent processing. This may provide for a (limited) form of ‘downward causation’ in which our conscious experiences (assuming these line up with the overall winning hypotheses) play a special functional role in the unfolding of future thoughts and actions.

^12^I do not think, however, that the basic use of top-down probabilistic expectations should in general be treated (as a few commentators seemed to suppose) as an implausibly intellectualist route to adaptive response. Instead, the ability to process incoming information in ways determined by the online extraction of statistical patterns seems pervasive, and its use in recurrent processing may represent a fundamental biological strategy for dealing with limited information, uncertainty, and a changing world.
